# Open-chest versus closed-chest cardiopulmonary resuscitation in trauma patients with signs of life upon hospital arrival: a retrospective multicenter study

**DOI:** 10.1186/s13054-020-03259-w

**Published:** 2020-09-01

**Authors:** Akira Endo, Mitsuaki Kojima, Zhi-Jie Hong, Yasuhiro Otomo, Raul Coimbra

**Affiliations:** 1 Trauma and Acute Critical Care Center, Tokyo Medical and Dental University Hospital of Medicine, 1-5-45 Yushima, Bunkyo-ku, Tokyo, Japan; 2 Emergency and Critical Care Medicine, Tokyo Women’s Medical University Medical Center East, 2-1-10 Nishiogu, Arakawa-ku, Tokyo, Japan; 3 Riverside University Health System, Comparative Effectiveness and Clinical Outcomes Research Center, 26520 Cactus Avenue, CPC Suite 102-5, Moreno Valley, CA 92555 USA; 4 Division of Traumatology, Department of Surgery, Tri-Service General Hospital, National Defense Medical Center, Taipei, Taiwan, Republic of China

**Keywords:** Polytrauma, Resuscitation, Resuscitative thoracotomy, Cardiac arrest, Shock, Registry, Open-chest cardiopulmonary resuscitation, Closed-chest cardiopulmonary resuscitation

## Abstract

**Background:**

The effectiveness and indications of open-chest cardiopulmonary resuscitation (OCCPR) have been still debatable. Although current guidelines state that the presence of signs of life (SOL) is an indication for OCCPR, scientific evidence corroborating this recommendation has been scarce. This study aimed to compare the effectiveness of OCCPR to closed-chest cardiopulmonary resuscitation (CCCPR) in severe trauma patients with SOL upon arrival at the emergency department (ED).

**Methods:**

A retrospective cohort study analyzing data from the Trauma Quality Improvement Program (TQIP) database, a nationwide trauma registry in the USA, between 2010 and 2016 was conducted. Severe trauma patients who had SOL upon arrival at the hospital and received cardiopulmonary resuscitation within the first 6 h of ED admission were identified. Survival to hospital discharge was evaluated using logistic regression analysis, instrumental variable analysis, and propensity score matching analysis adjusting for potential confounders.

**Results:**

A total of 2682 patients (OCCPR 1032; CCCPR 1650) were evaluated; of those 157 patients (15.2%) in the OCCPR group and 193 patients (11.7%) in the CCCPR group survived. OCCPR was significantly associated with higher survival to hospital discharge in both the logistic regression analysis (adjusted odds ratio [95% confidence interval] = 1.99 [1.42–2.79], *p* <  0.001) and the instrumental variable analysis (adjusted odds ratio [95% confidence interval] = 1.16 [1.02–1.31], *p* = 0.021). In the propensity score matching analysis, 531 matched pairs were generated, and the OCCPR group still showed significantly higher survival at hospital discharge (89 patients [16.8%] in the OCCPR group vs 58 patients [10.9%] in the CCCPR group; odds ratio [95% confidence interval] = 1.66 [1.13–2.42], *p* = 0.009).

**Conclusions:**

Compared to CCCPR, OCCPR was associated with significantly higher survival at hospital discharge in severe trauma patients with SOL upon ED arrival. Further studies to confirm these results and to assess long-term neurologic outcomes are needed.

## Background

Open-chest cardiopulmonary resuscitation (OCCPR) came into use in the USA in the late 1800s as the salvage maneuver following cardiac arrest. It simultaneously includes control of infra diaphragmatic hemorrhage by cross-clamping of the descending thoracic aorta, in addition to direct cardiac massage, when necessary [1, 2]. Because it is impractical to conduct randomized controlled trials comparing between OCCPR and closed-chest cardiopulmonary resuscitation (CCCPR) in traumatic cardiac arrest cases due to ethical reasons, the therapeutic impact of OCCPR has only been evaluated in observational and cohort studies [3–5]. However, the survival benefit of OCCPR compared to CCCPR has not been clearly determined even in a recent meta-analysis [6].

Because of the poor cost-effectiveness and potential infectious risks to medical staff [7, 8], in addition to the aforementioned evidence, recent guidelines have restricted the indications for the use of resuscitative thoracotomy (RT) [2, 9, 10]. The indications for RT in those guidelines are generally based upon a positive finding of signs of life (SOL: detectable blood pressure, respiratory or motor effort, cardiac electrical activity, or pupillary activity) and the time from onset of cardiac arrest because patient survival is believed to be rare after more than 15 min of cardiopulmonary resuscitation [11]. However, most of the recommendations have been based on descriptive studies of small sample sizes or expert opinions and not based on a large-scale cohort study evaluating the comparative effectiveness of OCCPR to CCCPR.

In the present study, we evaluated the effectiveness of OCCPR, compared to CCCPR, in trauma patients who had SOL upon emergency department (ED) admission, based on the hypothesis that OCCPR is associated with better survival outcomes than CCCPR in those patients.

## Methods

### Study design and settings

A retrospective cohort study analyzing data of the Trauma Quality Improvement Program (TQIP) database between 2010 and 2016 was conducted. The TQIP database is a subset of the National Trauma Databank of the American College of Surgeons Committee on Trauma, which stores data of patients aged more than 15 years and suffered severe injury, defined by abbreviated injury scale (AIS) ≥ 3. At the end of 2016, more than 700 level 1 and level 2 trauma centers participated in the TQIP database. Trained specialists abstracted more than 100 variables for each patient as well as information on the treating hospital. Details in the TQIP database are available at https://www.facs.org/quality-programs/trauma/tqp/center-programs/tqip. Survival to hospital discharge of patients who received OCCPR was compared to that of patients who received CCCPR only.

This study complied with the principles of the 1964 Helsinki Declaration and its later amendments. The study and its protocols were in compliance with the institutional review board of Riverside University Health System—Comparative Effectiveness and Clinical Outcomes Research Center (approval number: 1636962). The requirement for informed consent for each patient was waived based on the use of anonymized patient and hospital data.

### Study population

We included trauma patients who met the following criteria: (i) presence of SOL upon hospital arrival and (ii) received OCCPR or CCCPR within 6 h of hospital arrival. Due to the inclusion criteria of the TQIP database, patients younger than 16 years were not included. We also excluded patients who had a nonsurvivable injury defined by 6 points in AIS, patients without exact information on injury mechanism, or patients without exact information on SOL upon ED arrival. The patients were divided into the OCCPR and the CCCPR groups, and their outcomes were compared between the two groups.

### Data collection

The following variables were collected from the TQIP database: age, gender, insurance type, vital signs (systolic blood pressure, heart rate, respiratory rate), Glasgow Coma Scale (GCS), body temperature, presence or absence of SOL upon ED arrival, year of injury, injury mechanism (i.e., blunt or penetrating), AIS in each body region, Injury Severity Score (ISS), total prehospital transport time, implementation of OCCPR or CCCPR and their timing (recorded on an hour basis after ED arrival), length of hospital stay, and survival status at hospital discharge. Hospital information regarding trauma center level, teaching status, and number of trauma surgeons was also collected.

### Outcome measure and cohort definitions

The study outcome was defined as survival to hospital discharge. The OCCPR group was defined as patients who received OCCPR within the first 6 h of ED arrival with or without CCCPR prior to OCCPR, considering the clinical importance of the first 6 h after injury [12]. Meanwhile, the CCCPR group was defined as patients who received CCCPR only, within the first 6 h of ED arrival. Patients who had both codes for OCCPR and CCCPR were classified into the OCCPR group because executing CCCPR is common during the preparation for OCCPR, but the reverse cannot be true from a practical clinical perspective. The implementation of OCCPR and CCCPR was identified using the procedure codes of the International Classification of Diseases 9th Revision Clinical Modification (ICD-9-CM) 37.91 and 99.63, respectively.

### Statistical analysis

Missing values were treated by multiple imputation using chained equations with 10 iterations and creation of 15 datasets, based on the assumption of missing at random in missing mechanism as well as previous studies using the TQIP database [13, 14].

Three statistical models were used for analyses: (i) logistic regression analysis, (ii) instrumental variable analysis, and (iii) propensity score matching analysis. Covariates used for case-mix classification, used in the logistic regression model and in the instrumental variable model, included patient age, gender, insurance type, year of injury, injury mechanism (i.e., penetrating or blunt), vital signs upon ED arrival (systolic blood pressure, heart rate, respiratory rate), GCS, and body temperature at ED arrival, maximum AIS by body region, ISS, total prehospital transport time, and hospital characteristics (American College of Surgeons verification level and teaching status). The variables were selected based on clinical perspective. Issues with variable multicollinearity were assessed using variance inflation factor (VIF) analysis, and the tolerance value was set at less than 2. In the instrumental variable analysis, which is an established technique to control unmeasured confounding in non-randomized data [15], the number of trauma surgeons in a hospital (categorized by whether more or less than 8 surgeons) was used as the instrumental variable. The cut-off value of the instrumental variable was determined according to the categorization in the number of trauma surgeons in the TQIP database. This approach was conducted using a two-stage least-squares regression adjusted by the aforementioned variables, based on the null hypothesis that there was no association between the number of trauma surgeons in a hospital and the actual implementation of OCCPR. A partial *F* test was conducted to assess an issue of weak instruments, and a value of *F*-statistic more than 10 was regarded as acceptable.

Considering the heterogeneity in the characteristics between the OCCPR and CCCPR groups, we also performed a propensity score matching analysis [16]. In this analysis, a logistic regression model was applied to estimate the propensity score to predict OCCPR in each patient using the variables mentioned above. Propensity score matching extracted 1:1 matched pairs from the OCCPR and CCCPR groups. Match balance between the two groups was assessed using the absolute standardized mean difference (ASMD), and values of less than 0.1 were considered acceptable. The caliper width was set as the standard deviation of the logit-transformed propensity score multiplied by 0.002 to achieve well match balance between two groups. The chi-square test was used for intergroup comparison in the propensity score-matched cohort. Furthermore, cumulative incidence curves for the in-hospital mortality in the propensity score-matched subjects were constructed. The Fine and Gray test was used to estimate the subdistribution hazard ratio for in-hospital mortality, considering the competing risk between in-hospital mortality and survival discharge [17].

Since the information on survival outcome was lacking in some patients, in addition to the multiple imputation method, we performed sensitivity analyses in which the outcome was imputed based on the most optimistic and pessimistic scenarios, where all the missing information on survival outcome was assumed as survival or death, respectively. The aforementioned logistic regression model was applied in these sensitivity analyses.

Descriptive statistics were used to display categorical variables as counts and percentages, and numeric or ordered variables as medians and 25th–75th percentiles, after pooling all the imputed datasets into one dataset. Predictive statistics were used to display the estimators as point estimation and 95% confidence intervals (CIs) integrated across the imputed datasets, based on Rubin’s rule [18]. The level of significance was defined as *p* <  0.05 for all statistical analyses. All the analyses were performed using R 3.5.3 (R Foundation for Statistical Computing, Vienna, Austria) with add-on packages of “mice [19]” for multiple imputation, “Matching [20]” for propensity score matching, “AER [21]” for instrumental variable analysis, and “cmprsk [22]” for the Fine and Gray test.

## Results

**Fig. 1:**
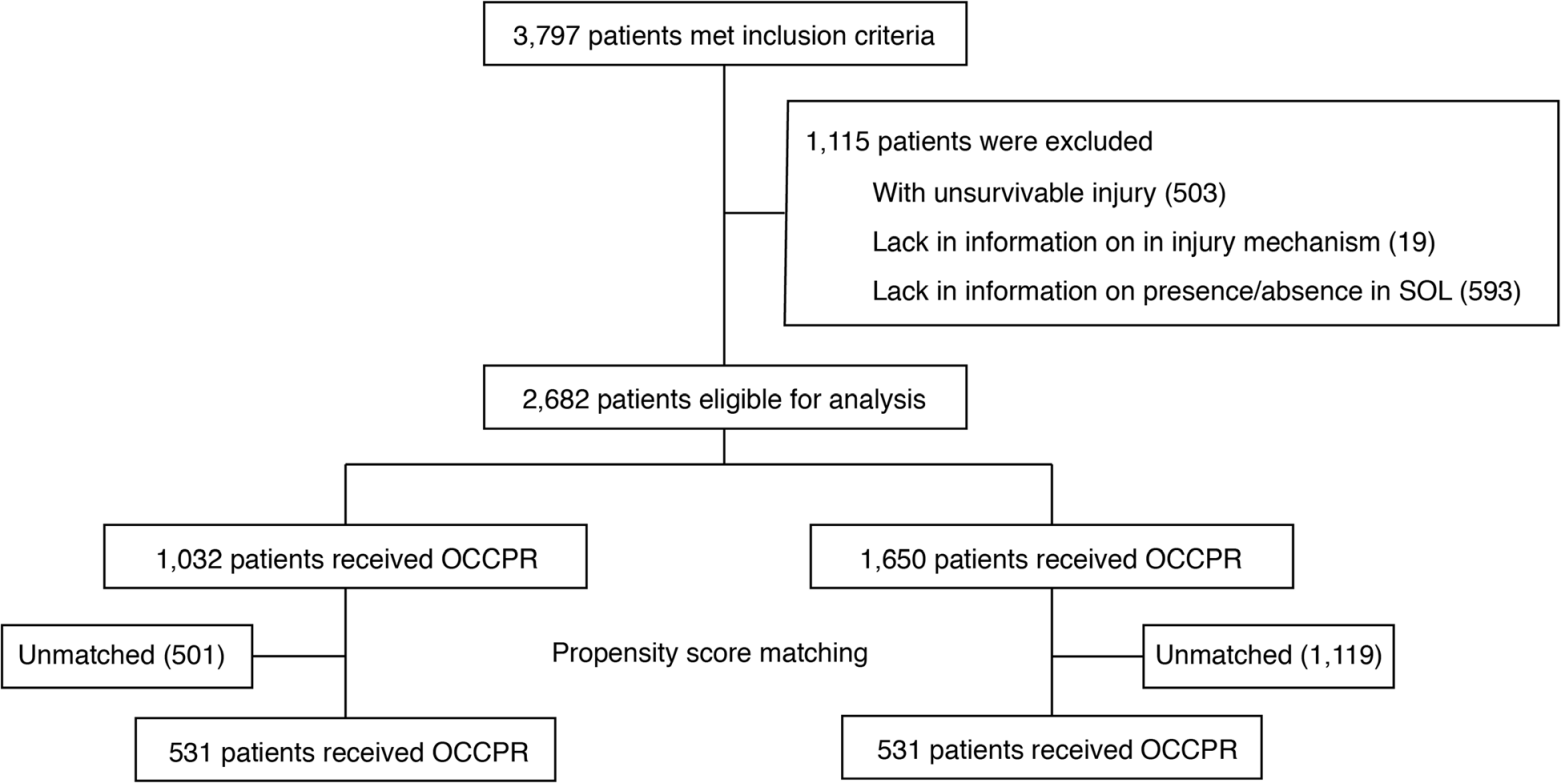
Flow diagram of patient selection. Abbreviations: SOL, signs of life; OCCPR, open-chest cardiopulmonary resuscitation; CCCPR, closed-chest cardiopulmonary resuscitation

The flow diagram of patients is shown in Fig. 1. A total of 2682 patients (OCCPR 1032; CCCPR 1650) were eligible for analysis. The characteristics of naïve data, including the proportion of missing values, are shown in Additional file 1. The major baseline characteristics of the patients of the multiply imputed cohort are shown in Table 1. The OCCPR group was older than the CCCPR group (median [25th–75th percentiles] = 45 [28, 63] and 32 [23, 49], respectively), and the proportion of blunt trauma was higher in the OCCPR group (71.8%) than in the CCCPR group (42.7%).

**Table 1:** Baseline characteristics of the patients in the multiply imputed dataset (major variables)

Variables	OCCPR (*n* = 1032)	CCCPR (*n* = 1650)	ASMD
Age, years old, median [IQR]	45 [28, 63]	32 [23, 49]	0.45
Gender, female, *n* (%)	267 (25.9)	311 (18.8)	0.17
Type of injury			
Blunt	741 (71.8)	705 (42.7)	0.61
Penetrating	291 (28.2)	945 (57.3)	0.61
Total prehospital transport time, min, median [IQR]	48 [32, 88]	35 [25. 59]	0.15
Transfer from another hospital, yes, *n* (%)	110 (10.7)	117 (7.1)	0.13
Highest AIS score per body region, median [IQR]			
Head	3 [0, 5]	0 [0, 1]	0.79
Face	0 [0, 1]	0 [0, 0]	0.28
Neck	0 [0, 0]	0 [0, 0]	0.02
Chest	3 [0, 4]	3 [3, 4]	0.45
Abdomen	0 [0, 3]	3 [0, 4]	0.43
Spine	0 [0, 2]	0 [0, 0]	0.22
Upper extremities	0 [0, 2]	0 [0, 1]	0.02
Pelvis and lower extremities	0 [0, 3]	0 [0, 2]	0.17
Skin/superficial	0 [0, 0]	0 [0, 0]	0.00
Injury Severity Score	26 [19, 35]	26 [20, 36]	0.10
Systolic blood pressure, mmHg, median [IQR]	100 [72, 132]	97 [69, 127]	0.09
Heart rate, bpm, median [IQR]	103 [70, 129]	110 [77, 133]	0.12
Respiratory rate, bpm, median [IQR]	16 [0, 22]	17 [8, 24]	0.11
Body temperature, °C, median [IQR]	36.0 [35.1, 36.5]	36.0 [35.2, 36.5]	0.00
Glasgow Coma Scale, median [IQR]	3 [3, 9]	3 [3, 13]	0.22
Time from ED arrival to OCCPR, hours, median [IQR]	1 [0, 1]	–	–
Time from ED arrival to CCCPR, hours, median [IQR]	0 [0, 1]	0 [0, 1]	0.25

*Abbreviations: OCCPR* open-chest cardiopulmonary resuscitation, *OCCPR* closed-chest cardiopulmonary resuscitation, *ASMD* absolute standardized mean difference, *IQR* interquartile range, *AIS* abbreviated injury scale, *ED* emergency department

Detailed baseline characteristics of the cohort are shown in Additional file 2. The characteristics of the hospitals of the cohort are shown in Table 2. The number of trauma surgeons was higher in the OCCPR group than in the CCCPR group. In total, 157 patients (15.2%) in the OCCPR group and 193 patients (11.7%) in the CCCPR group survived to hospital discharge (crude odds ratio [95% CI] = 1.35 [1.06–1.73], *p* = 0.017). The median length of hospital stay [25th–75th percentiles] among the survivors in the OCCPR group and the CCCPR group was 18 days [6–35] and 19 days [10–32], respectively.

**Table 2:** Baseline characteristics of the hospitals in the multiply imputed dataset

Variables	OCCPR (*n* = 1032)	CCCPR (*n* = 1650)	ASMD
ACS trauma center level, *n* (%)			
I	807 (78.2)	1248 (75.6)	0.06
II	225 (21.8)	402 (24.4)	0.06
Teaching status, *n* (%)			
University	719 (69.7)	1097 (66.5)	0.07
Community	226 (21.9)	470 (28.5)	0.15
Non-teaching	87 (8.4)	83 (5.0)	0.14
Number of trauma surgeons, > 8, *n* (%)	400 (38.8)	445 (27.0)	0.25

*Abbreviations: OCCPR* open-chest cardiopulmonary resuscitation, *OCCPR* closed-chest cardiopulmonary resuscitation, *ASMD* absolute standardized mean difference, *ACS* American College of Surgeons

In the logistic regression analysis, all of the VIFs of used variables were lower than 2, which eliminated the issue of multicollinearity in our model. OCCPR was significantly associated with higher survival at discharge in the logistic regression analysis (adjusted odds ratio [95% CI] = 1.99 [1.42–2.79], *p* <  0.001). In the instrumental variable analysis, the linear regression analysis demonstrated that there was a significant increase in the likelihood of OCCPR implementation according to the number of trauma surgeons (adjusted odds ratio [95% CI] = 1.11 [1.08–1.15], *p* <  0.001, *F*-statistic = 28.9). Therefore, the null hypothesis that there was no association between the number of trauma surgeons and the actual implementation of OCCPR was rejected. However, survival to hospital discharge was not significantly affected by the instrumental variable in the linear regression analysis adjusted by OCCPR (adjusted odds ratio [95% CI] = 1.01 [0.98–1.04], *p* = 0.485). Therefore, the variable “number of trauma surgeons in a hospital” satisfied the requirements of the instrumental variable. The two-stage least-squares analysis with this instrumental variable also demonstrated significantly higher survival associated with OCCPR (adjusted odds ratio [95% CI] = 1.16 [1.02–1.31], *p* = 0.021).

In the propensity score matching analysis, through the one-to-one matching process, 531 matched pairs were selected. The major baseline characteristics of the patients in the multiply imputed and propensity score-matched cohort are shown in Table 3. The detailed baseline characteristics of the cohort are shown in Additional file 3. The characteristics of the hospitals of the cohort are shown in Table 4.

**Table 3:** Baseline characteristics of the patients of the multiply imputed and propensity score-matched dataset (major matched variables)

Variables	OCCPR (*n* = 531)	CCCPR (*n* = 531)	ASMD
Age, years old, median [IQR]	39 [26, 56]	40 [25, 56]	< 0.01
Gender, female, *n* (%)	127 (23.9)	127 (23.9)	< 0.01
Type of injury			
Blunt	339 (63.8)	342 (64.4)	0.01
Penetrating	192 (36.2)	189 (35.6)	0.01
Total prehospital transport time, min, median [IQR]	44 [30, 73]	39 [28, 69]	0.02
Transfer from another hospital, yes, *n* (%)	47 (8.9)	47 (8.9)	< 0.01
Highest AIS score per body region, median [IQR]			
Head	0 [0, 3]	0 [0, 4]	0.07
Face	0 [0, 1]	0 [0, 1]	< 0.01
Neck	0 [0, 0]	0 [0, 0]	0.04
Chest	3 [3, 4]	3 [3, 4]	0.05
Abdomen	2 [0, 3]	2 [0, 4]	0.07
Spine	0 [0, 0]	0 [0, 2]	0.03
Upper extremities	0 [0, 2]	0 [0, 2]	0.03
Pelvis and lower extremities	0 [0, 3]	0 [0, 3]	< 0.01
Skin/superficial	0 [0, 0]	0 [0, 0]	0.03
Injury Severity Score	26 [19, 35]	27 [20, 38]	0.08
Systolic blood pressure, mmHg, median [IQR]	92 [67, 127]	96 [69, 130]	0.05
Heart rate, bpm, median [IQR]	107 [71, 130]	106 [75, 130]	0.03
Respiratory rate, bpm, median [IQR]	16 [0, 22]	16 [8, 24]	0.09
Body temperature, °C, median [IQR]	36.0 [35.0, 36.5]	36.0 [35.3, 36.5]	0.04
Glasgow Coma Scale, median [IQR]	3 [3, 11]	3 [3, 12]	0.06

*Abbreviations: OCCPR* open-chest cardiopulmonary resuscitation, *OCCPR* closed-chest cardiopulmonary resuscitation, *ASMD* absolute standardized mean difference, *IQR* interquartile range, *AIS* abbreviated injury scale, *ED* emergency department, *ACS* American College of Surgeons

**Table 4:** Baseline characteristics of the hospitals of the multiply imputed and propensity score-matched dataset

Variables	OCCPR (*n* = 531)	CCCPR (*n* = 531)	ASMD
ACS trauma center level, *n* (%)			
I	424 (79.9)	416 (78.3)	0.04
II	107 (20.1)	115 (21.7)	0.04
Teaching status, *n* (%)			
University	377 (71.0)	362 (68.2)	0.06
Community	125 (23.5)	136 (25.6)	0.05
Non-teaching	29 (5.5)	33 (6.2)	0.03

*Abbreviations: OCCPR* open-chest cardiopulmonary resuscitation, *OCCPR* closed-chest cardiopulmonary resuscitation, *ASMD* absolute standardized mean difference, *ACS* American College of Surgeons

All the values of ASMDs in the adjusted variables were less than 0.1, indicating a well-matched balance (Tables 3 and 4 and Additional file 3). In the propensity score-matched cohort, the median age [25th–75th percentiles] was 40 years old [25–56], and 254 patients (23.9%) were female. Penetrating injury was observed in 381 patients (35.9%). In total, 89 patients (16.8%) in the OCCPR group and 58 patients (10.9%) in the CCCPR group survived to hospital discharge. The OCCPR group showed significantly higher survival also in the propensity score-matched cohort (odds ratio [95% CI] = 1.66 [1.13–2.42], *p* = 0.009). The results of these three analyses are summarized in Table 5.

**Table 5:** Comparative effectiveness of OCCPR, compared to CCCPR, for survival to hospital discharge evaluated by the logistic regression analysis, instrumental variable analysis, and propensity score matching analysis

Models	Number of survivors		Adjusted odds ratio [95% confidence interval]	*p* value
	OCCPR	CCCPR		
Logistic regression analysis	157/1032 (15.2%)	293/1650 (11.7%)	1.99 [1.42–2.79]	< 0.001
Instrumental variable analysis	157/1032 (15.2%)	293/1650 (11.7%)	1.16 [1.02–1.31]	0.021
Propensity score matching analysis	89/531 (16.8%)	58/531 (10.9%)	1.66 [1.13–2.42]	0.009

*Abbreviations: OCCPR* open-chest cardiopulmonary resuscitation, *CCCPR* closed-chest cardiopulmonary resuscitation

**Fig. 2:**
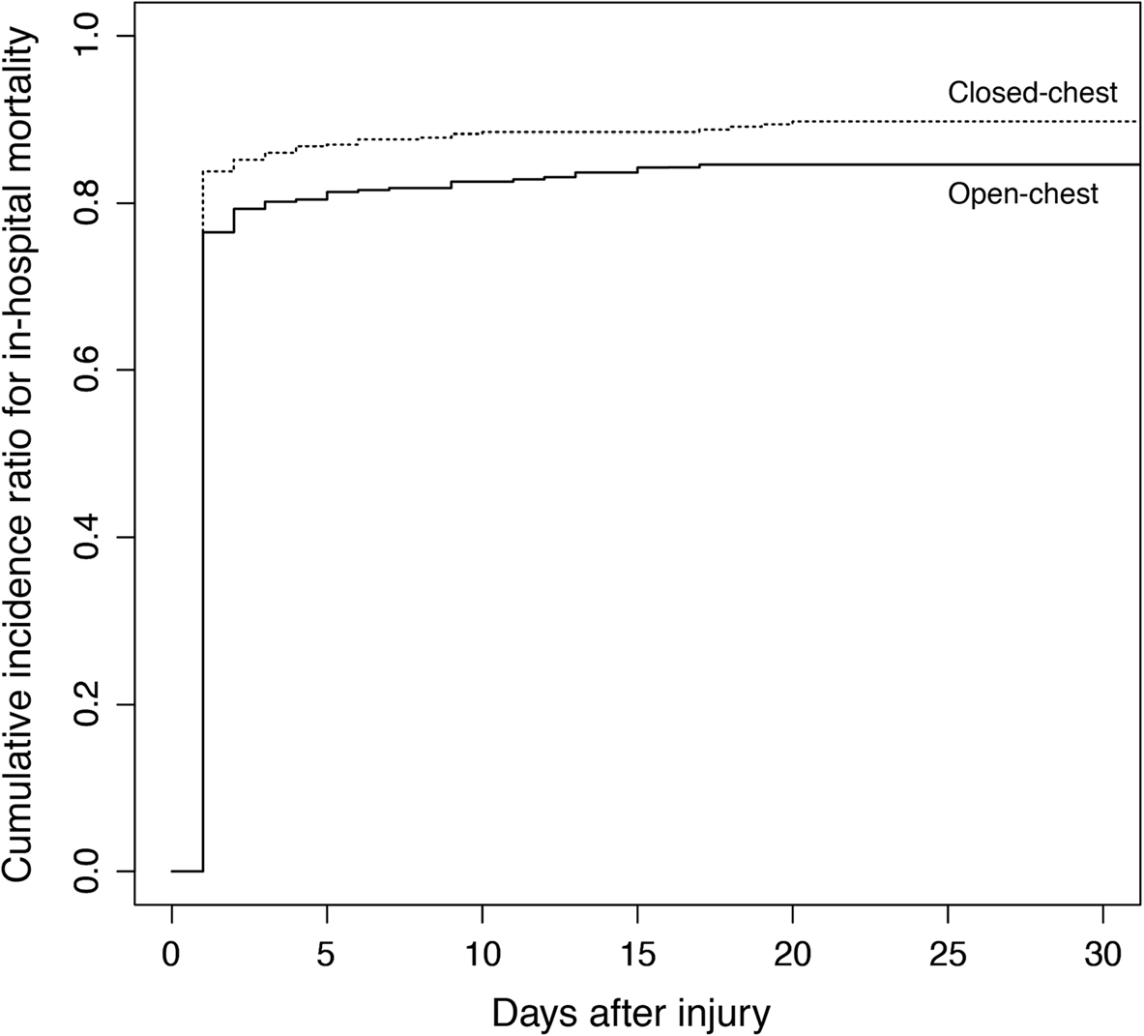
Cumulative incidence curves for in-hospital mortality in the propensity score-matched subjects

The cumulative incidence curve of in-hospital mortality until 30 days after admission in the propensity score-matched subjects is shown in Fig. 2. A marked difference in mortality was observed, particularly in the first day of admission. The Fine and Gray test showed that OCCPR was significantly associated with lower in-hospital mortality (subdistribution hazard ratio [95% confidence intervals] = 0.92 [0.86–0.98], *p* = 0.009).

In the sensitivity analyses, where all the missing information on the outcome was assumed as survival, as in the original analysis, OCCPR was significantly associated with higher survival at hospital discharge (adjusted odds ratio [95% CI] = 2.87 [2.32–3.55], *p* < 0.001). The similar result was observed in the analysis where all the missing information on the outcome was assumed as death (adjusted odds ratio [95% CI] = 1.80 [1.31–2.47], *p* < 0.001).

## Discussion

In the present retrospective observational study, we evaluated the survival benefit of OCCPR compared to CCCPR in trauma patients who had SOL upon ED arrival. All of the three statistical models indicated a significant survival benefit of OCCPR. To the best of our knowledge, this is the first study to validate the indications for OCCPR by using a large-scale dataset and demonstrates a more favorable survival outcome of OCCPR compared to those of CCCPR.

OCCPR has several theoretical advantages over CCCPR in trauma patients’ resuscitation efforts [1, 2]. It would be difficult to achieve sufficient systemic blood perfusion by CCCPR in cases of multiple rib fractures and flail chest due to the reduced rib cage compliance. Cross-clamping of the descending thoracic aorta, which is usually combined with OCCPR, enables maintenance of cerebral/coronary blood perfusion and temporal control of infra diaphragmatic hemorrhage. However, previous studies failed to demonstrate the advantages of OCCPR compared to CCCPR [3, 4]. The discrepancy might be mainly explained by the difference in the study populations. The evaluated population in the present study was limited to patients who had SOL upon ED arrival; however, 41.2% of evaluated patients in a previous study [3] had cardiac arrest upon ED arrival. Moreover, 46.1% of the present study cohort were victims of penetrating mechanism, whereas all of the analyzed patients in a previous study were injured by blunt mechanisms [3]. The differences in the prehospital medical system and the distribution of trauma center as well as their coverage area might also have influenced the results of the studies. While the prehospital transport time was longer in the present study than that in a previous Japanese study [3], paramedics in the USA are allowed to provide a variety of medical interventions compared to their Japanese counterparts. The differences in these factors might have largely affected the survival rate observed in the present study (13.0% in the overall study cohort), which was higher than that of previously reported [23–26].

Joseph et al. [27] reviewed patients who underwent exploratory thoracotomy (ICD-9-CM, 34.02) within the first hour of hospital admission using the TQIP database and reported 9.6% survival rate. However, their study included patients without SOL on hospital arrival (28.6% of the analyzed cohort). Furthermore, OCCPR is only a part of exploratory thoracotomy and is not always performed during the procedure [28, 29]. In contrast, the present study used more strict definitions for the interventions (i.e., OCCPR or CCCPR), and this might explain some differences in patient background and the results between their research and ours.

The duration from the time of cardiac arrest to OCCPR implementation is one of the important indicators in the recommendation of the current guidelines [2, 9, 10]. Actually, Yamamoto et al. [30] reported that a short-duration CCCPR time before OCCPR was associated with significantly higher recovery of spontaneous circulation (ROSC) rates. However, in a retrospective database analysis, there remained a major concern that OCCPR was performed only in patients who could not achieve ROSC by CCCPR alone [6]. This bias is generally associated with worse outcomes in patients who received OCCPR. Although the present study could not overcome this bias due to the nature of the TQIP database in which detailed time course data in minutes were not available, considering the direction of this bias, the results suggested that the presence of SOL might be one of the strong indicators for the implementation of OCCPR. However, further studies accounting for the detailed time course are necessary to control this bias.

The strength of the present study was that a large number of patients were analyzed using several rigorous statistical approaches. The data on the presence or absence of SOL, one of the indications for RT in the current guidelines [2, 9, 10], were available in the TQIP database. Moreover, the procedures of OCCPR and CCCPR could be clearly identified. However, this study also had some limitations. Since this was a retrospective registry-based study, the issue of residual confounding was unavoidable. The indication for OCCPR cannot be fully explained by registry variables. Although we made the best effort to overcome this bias by using an instrumental variable model, specifying an ideal instrumental variable is challenging in a retrospective registry-based study because there is no established method to verify the inexistence of the pathway from the instrumental variable to the outcome. The issue of potential uncertainty in the registry data, including ICD-9-CM procedure codes, was also one of the limitations of this study. There were missing data to some degree, including the study outcome, to which we used a multiple imputation method. Detailed information on time course in minutes was unavailable, as described above. Data on neurological outcomes, the ultimate outcome measure of resuscitation treatments, were also unavailable in the TQIP database. Finally, data on patients younger than 16 years were not available, which prevented us from evaluating this age group.

Despite these limitations, this was the first well-designed large-scale study that corroborated one of the indications in the current recommendations for the use of OCCPR in trauma patients. The results of our study showed a significant association between OCCPR implementation and favorable survival outcome in trauma patients who had SOL on ED arrival. From these results, it would be expected that patient survival increases by maintaining SOL until ED arrival combined with subsequent OCCPR. Further studies from other countries are necessary to confirm the generalizability of our findings. In addition, the influence of time from onset of cardiac arrest, which is another major criterion in the current trauma guideline, should also be statistically evaluated in future studies to identify the best possible candidates for OCCPR.

## Conclusions

OCCPR was associated with significantly higher survival to hospital discharge than CCCPR in trauma patients with SOL upon ED arrival. Further studies to confirm these results and to assess neurological outcomes, taking detailed time course into account, are required.

## Supplementary information

**Additional file 1: Table S1** Baseline characteristics and proportion of missing data in naïve dataset.

**Additional file 2: Table S2** Baseline characteristics of the patients in the multiply imputed dataset (all variables).

**Additional file 3.** Baseline characteristics of the patients in the multiply imputed and propensity score-matched dataset (all the matched variables).

## Data Availability

All data used in the present study are available from the American College of Surgeons Trauma Quality Improvement Program.

## Abbreviations

AIS: Abbreviated injury scale
ASMD: Absolute standardized mean difference
CCCPR: Closed-chest cardiopulmonary resuscitation
CI: Confidence interval
CPR: Cardiopulmonary resuscitation
ED: Emergency department
GCS: Glasgow Coma Scale
ICD-9-CM: International Classification of Diseases 9th Revision Clinical Modification
ISS: Injury Severity Score
OCCPR: Open-chest cardiopulmonary resuscitation
ROSC: Recovery of spontaneous circulation
RT: Resuscitative thoracotomy
SOL: Signs of life
TQIP: Trauma Quality Improvement Program
VIF: Variance inflation factor
