# Supplementary material for: Open-chest versus closed-chest cardiopulmonary resuscitation in trauma patients with signs of life upon hospital arrival: a retrospective multicenter study
Source: Crit Care. 2020 Sep 1;24:541. doi: 10.1186/s13054-020-03259-w (PMC7465718; doi:10.1186/s13054-020-03259-w)
Supplement: Supplementary file 1 — Additional file 1: Table S1 Baseline characteristics and proportion of missing data in naïve dataset. [file 13054_2020_3259_MOESM1_ESM.docx]

| **Table S1. Baseline characteristics and proportion of missing data in naïve dataset** | | | | | |
| --- | --- | --- | --- | --- | --- |
| Variables | | OCCPR  (n = 1,032) | | CCCPR  (n = 1,650) | |
|  |  | Registered data | Missing, n (%) | Registered data | Missing, n (%) |
| Patient characteristics | | | | | |
| Age, years old, median [IQR] | | 45 [28, 63] | 0 (0) | 32 [23, 49] | 0 (0) |
| Gender, female, n (%) | | 267 (25.9) | 0 (0) | 311 (18.8) | 1 (0.1) |
| Insurance type, n (%) | |  | 68 (6.6) |  | 77 (4.7) |
|  | Blue Cross/Blue Shield | 32 (3.1) |  | 47 (2.8) |  |
|  | Medicaid | 74 (7.2) |  | 222 (13.5) |  |
|  | Medicare | 166 (16.1) |  | 86 (5.2) |  |
|  | No Fault Automobile | 54 (5.2) |  | 99 (6.0) |  |
|  | Not Billed (for any reason) | 2 (0.2) |  | 21 (1.3) |  |
|  | Other Government | 40 (3.9) |  | 56 (3.4) |  |
|  | Private/Commercial Insurance | 197 (19.1) |  | 259 (15.7) |  |
|  | Self Pay | 353 (34.2) |  | 716 (43.4) |  |
|  | Workers Compensation | 13 (1.3) |  | 22 (1.3) |  |
|  | Others | 33 (3.2) |  | 45 (2.7) |  |
| Year of injury, n (%) | |  | 3 (0.3) |  | 1 (0.1) |
|  | 2010 | 72 (7.0) |  | 41 (2.5) |  |
|  | 2011 | 174 (16.9) |  | 217 (13.2) |  |
|  | 2012 | 148 (14.3) |  | 262 (15.9) |  |
|  | 2013 | 153 (14.8) |  | 258 (15.6) |  |
|  | 2014 | 168 (16.3) |  | 315 (19.1) |  |
|  | 2015 | 208 (20.2) |  | 479 (29.0) |  |
|  | 2016 | 106 (10.3) |  | 77 (4.7) |  |
| Type of injury | |  | 0 (0) |  | 0 (0) |
|  | Blunt | 741 (71.8) |  | 705 (42.7) |  |
|  | Penetrating | 291 (28.2) |  | 945 (57.3) |  |
| Total prehospital transport time, min, median [IQR] | | 48 [32, 87] | 73 (7.1) | 35 [25, 58] | 220 (13.3) |
| Transfer from another hospital, Yes, n (%) | | 110 (10.7) | 0 (0) | 117 (7.1) | 0 (0) |
| Highest AIS score per body region, median [IQR] | |  |  |  |  |
|  | Head | 3 [0, 5] | 0 (0) | 0 [0, 1] | 0 (0) |
|  | Face | 0 [0, 1] | 0 (0) | 0 [0, 0] | 2 (0.1) |
|  | Neck | 0 [0, 0] | 0 (0) | 0 [0, 0] | 0 (0) |
|  | Chest | 3 [0, 4] | 0 (0) | 3 [3, 4] | 0 (0) |
|  | Abdomen | 0 [0, 3] | 0 (0) | 3 [0, 4] | 0 (0) |
|  | Spine | 0 [0, 2] | 0 (0) | 0 [0, 0] | 0 (0) |
|  | Upper extremities | 0 [0, 2] | 1 (0.1) | 0 [0, 1] | 1 (0.1) |
|  | Pelvis and lower extremities | 0 [0, 3] | 1 (0.1) | 0 [0, 2] | 2 (0.1) |
|  | Skin/Superficial | 0 [0, 0] | 0 (0) | 0 [0, 0] | 0 (0) |
| Injury Severity Score | | 26 [19, 35] | 2 (0.2) | 26 [21, 38] | 73 (4.4) |
| Systolic blood pressure, mmHg, median [IQR] | | 100 [72, 132] | 81 (7.8) | 96 [69, 127] | 199 (12.1) |
| Heart rate, bpm, median [IQR] | | 102 [70, 129] | 39 (3.8) | 111 [78, 133] | 120 (7.3) |
| Respiratory rate, bpm, median [IQR] | | 16, [0, 22] | 109 (10.6) | 18 [10, 24] | 293 (17.8) |
| Body temperature, ℃, median [IQR] | | 36.0 [35.3, 36.6] | 497 (48.2) | 36.0 [35.3, 36.6] | 1,057 (64.7) |
| Glasgow coma scale, median [IQR] | | 3 [3, 9] | 28 (2.7) | 3 [3, 13] | 70 (4.2) |
| Time from ED arrival to OCCPR, hour, median [IQR] | | 1 [0, 1] | 0 (0) | - | - |
| Time from ED arrival to CCCPR, hour, median [IQR] | | 0 [0, 1] | 943 (91.4) | 0 [0, 1] | 0 (0) |
| Length of hospital stay, days, median [IQR] | | 1 [1, 1] | 19 (1.8) | 1 [1, 1] | 7 (0.4) |
| Survival to hospital discharge, n (%) | | 130 (12.6) | 427 (41.4) | 182 (11.0) | 307 (18.6) |
| Hospital characteristics | | | | | |
| ACS trauma center level, n (%) | |  | 347 (33.6) |  | 510 (30.9) |
|  | I | 587 (56.9) |  | 876 (53.1) |  |
|  | II | 98 (9.5) |  | 264 (16.0) |  |
| Teaching status, n (%) | |  | 0 (0) |  | 0 (0) |
|  | University | 719 (69.7) |  | 1,097 (66.5) |  |
|  | Community | 226 (21.9) |  | 470 (28.5) |  |
|  | Non-teaching | 87 (8.4) |  | 83 (5.0) |  |
| Number of trauma surgeon, >8, n (%) | | 400 (38.8) | 0 (0) | 445 (27.0) | 0 (0) |
| Abbreviations: OCCPR, open-chest cardiopulmonary resuscitation; OCCPR, closed-chest cardiopulmonary resuscitation; IQR, interquartile range; AIS, abbreviated injury scale; ED, emergency department; ACS, American College of Surgeons. | | | | | |
