# Supplementary material for: Open-chest versus closed-chest cardiopulmonary resuscitation in trauma patients with signs of life upon hospital arrival: a retrospective multicenter study
Source: Crit Care. 2020 Sep 1;24:541. doi: 10.1186/s13054-020-03259-w (PMC7465718; doi:10.1186/s13054-020-03259-w)
Supplement: Supplementary file 2 — Additional file 2: Table S2 Baseline characteristics of the patients in the multiply imputed dataset (all variables). [file 13054_2020_3259_MOESM2_ESM.docx]

| **Table S2. Baseline characteristics of the patients in the multiply imputed dataset (all variables)** | | | | |
| --- | --- | --- | --- | --- |
| Variables | | OCCPR  (n = 1,032) | CCCPR  (n = 1,650) | ASMD |
| Age, years old, median [IQR] | | 45 [28, 63] | 32 [23, 49] | 0.45 |
| Gender, female, n (%) | | 267 (25.9) | 311 (18.8) | 0.17 |
| Insurance type, n (%) | |  |  |  |
|  | Blue Cross/Blue Shield | 34 (3.3) | 49 (2.9) | 0.02 |
|  | Medicaid | 84 (8.1) | 236 (14.3) | 0.20 |
|  | Medicare | 173 (16.8) | 92 (5.6) | 0.36 |
|  | No Fault Automobile | 59 (5.7) | 107 (6.5) | 0.03 |
|  | Not Billed (for any reason) | 3 (0.3) | 23 (1.4) | 0.12 |
|  | Other Government | 45 (4.4) | 60 (3.7) | 0.04 |
|  | Private/Commercial Insurance | 210 (20.4) | 268 (16.2) | 0.11 |
|  | Self Pay | 370 (35.9) | 742 (44.9) | 0.19 |
|  | Workers Compensation | 15 (1.5) | 24 (1.5) | <0.01 |
|  | Others | 37 (3.6) | 49 (2.9) | 0.04 |
| Year of injury, n (%) | |  |  |  |
|  | 2010 | 73 (7.0) | 41 (2.5) | 0.21 |
|  | 2011 | 174 (16.9) | 217 (13.2) | 0.10 |
|  | 2012 | 148 (14.4) | 262 (15.9) | 0.04 |
|  | 2013 | 153 (14.9) | 258 (15.6) | 0.02 |
|  | 2014 | 168 (16.3) | 315 (19.1) | 0.07 |
|  | 2015 | 209 (20.2) | 479 (29.0) | 0.21 |
|  | 2016 | 106 (10.3) | 77 (4.7) | 0.21 |
| Type of injury | |  |  |  |
|  | Blunt | 741 (71.8) | 705 (42.7) |  |
|  | Penetrating | 291 (28.2) | 945 (57.3) |  |
| Total prehospital transport time, min, median [IQR] | | 48 [32, 88] | 35 [25. 59] | 0.15 |
| Transfer from another hospital, Yes, n (%) | | 110 (10.7) | 117 (7.1) | 0.13 |
| Highest AIS score per body region, median [IQR] | |  |  |  |
|  | Head | 3 [0, 5] | 0 [0, 1] | 0.79 |
|  | Face | 0 [0, 1] | 0 [0, 0] | 0.28 |
|  | Neck | 0 [0, 0] | 0 [0, 0] | 0.02 |
|  | Chest | 3 [0, 4] | 3 [3, 4] | 0.45 |
|  | Abdomen | 0 [0, 3] | 3 [0, 4] | 0.43 |
|  | Spine | 0 [0, 2] | 0 [0, 0] | 0.22 |
|  | Upper extremities | 0 [0, 2] | 0 [0, 1] | 0.02 |
|  | Pelvis and lower extremities | 0 [0, 3] | 0 [0, 2] | 0.17 |
|  | Skin/Superficial | 0 [0, 0] | 0 [0, 0] | 0.00 |
| Injury Severity Score | | 26 [19, 35] | 26 [20, 36] | 0.10 |
| Systolic blood pressure, mmHg, median [IQR] | | 100 [72, 132] | 97 [69, 127] | 0.09 |
| Heart rate, bpm, median [IQR] | | 103 [70, 129] | 110 [77, 133] | 0.12 |
| Respiratory rate, bpm, median [IQR] | | 16 [0, 22] | 17 [8, 24] | 0.11 |
| Body temperature, ℃, median [IQR] | | 36.0 [35.1, 36.5] | 36.0 [35.2, 36.5] | 0.00 |
| Glasgow coma scale, median [IQR] | | 3 [3, 9] | 3 [3, 13] | 0.22 |
| Time from ED arrival to OCCPR, hour, median [IQR] | | 1 [0, 1] | – | – |
| Time from ED arrival to CCCPR, hour, median [IQR] | | 0 [0, 1] | 0 [0, 1] | 0.25 |
| Abbreviations: OCCPR, open-chest cardiopulmonary resuscitation; OCCPR, closed-chest cardiopulmonary resuscitation; ASMD, Absolute standardized mean difference; IQR, interquartile range; AIS, abbreviated injury scale; ED, emergency department | | | | |
