# Supplementary material for: Open-chest versus closed-chest cardiopulmonary resuscitation in trauma patients with signs of life upon hospital arrival: a retrospective multicenter study
Source: Crit Care. 2020 Sep 1;24:541. doi: 10.1186/s13054-020-03259-w (PMC7465718; doi:10.1186/s13054-020-03259-w)
Supplement: Supplementary file 3 — Additional file 3. Baseline characteristics of the patients in the multiply imputed and propensity score-matched dataset (all the matched variables). [file 13054_2020_3259_MOESM3_ESM.docx]

| **Table S3. Baseline characteristics of the patients in the multiply imputed and propensity score-matched dataset (all the matched variables)** | | | | |
| --- | --- | --- | --- | --- |
| Variables | | OCCPR  (n = 531) | CCCPR  (n = 531) | ASMD |
| Age, years old, median [IQR] | | 39 [26, 56] | 40 [25, 56] | < 0.01 |
| Gender, female, n (%) | | 127 (23.9) | 127 (23.9) | < 0.01 |
| Insurance type, n (%) | |  |  |  |
|  | Blue Cross/Blue Shield | 23 (4.4) | 19 (3.6) | 0.04 |
|  | Medicaid | 50 (9.5) | 65 (12.2) | 0.09 |
|  | Medicare | 44 (8.2) | 47 (8.8) | 0.02 |
|  | No Fault Automobile | 44 (8.2) | 43 (8.0) | 0.01 |
|  | Not Billed (for any reason) | 2 (0.3) | 4 (0.7) | 0.05 |
|  | Other Government | 24 (4.5) | 18 (3.4) | 0.06 |
|  | Private/Commercial Insurance | 103 (19.5) | 102 (19.2) | < 0.01 |
|  | Self Pay | 211 (39.8) | 200 (37.8) | 0.04 |
|  | Workers Compensation | 9 (1.8) | 11 (2.2) | 0.03 |
|  | Others | 20 (3.8) | 22 (4.2) | 0.02 |
| Year of injury, n (%) | |  |  |  |
|  | 2010 | 31 (5.8) | 22 (4.1) | 0.07 |
|  | 2011 | 100 (18.9) | 86 (16.2) | 0.07 |
|  | 2012 | 78 (14.7) | 74 (13.9) | 0.02 |
|  | 2013 | 77 (14.5) | 85 (16.0) | 0.04 |
|  | 2014 | 92 (17.4) | 87 (16.4) | 0.03 |
|  | 2015 | 126 (23.8) | 139 (26.2) | 0.05 |
|  | 2016 | 27 (4.9) | 32 (7.2) | 0.09 |
| Type of injury | |  |  |  |
|  | Blunt | 339 (63.8) | 342 (64.4) | 0.01 |
|  | Penetrating | 192 (36.2) | 189 (35.6) | 0.01 |
| Total prehospital transport time, min, median [IQR] | | 44 [30, 73] | 39 [28, 69] | 0.02 |
| Transfer from another hospital, Yes, n (%) | | 47 (8.9) | 47 (8.9) | < 0.01 |
| Highest AIS score per body region, median [IQR] | |  |  |  |
|  | Head | 0 [0, 3] | 0 [0, 4] | 0.07 |
|  | Face | 0 [0, 1] | 0 [0, 1] | < 0.01 |
|  | Neck | 0 [0, 0] | 0 [0, 0] | 0.04 |
|  | Chest | 3 [3, 4] | 3 [3, 4] | 0.05 |
|  | Abdomen | 2 [0, 3] | 2 [0, 4] | 0.07 |
|  | Spine | 0 [0, 0] | 0 [0, 2] | 0.03 |
|  | Upper extremities | 0 [0, 2] | 0 [0, 2] | 0.03 |
|  | Pelvis and lower extremities | 0 [0, 3] | 0 [0, 3] | < 0.01 |
|  | Skin/Superficial | 0 [0, 0] | 0 [0, 0] | 0.03 |
| Injury Severity Score | | 26 [19, 35] | 27 [20, 38] | 0.08 |
| Systolic blood pressure, mmHg, median [IQR] | | 92 [67, 127] | 96 [69, 130] | 0.05 |
| Heart rate, bpm, median [IQR] | | 107 [71, 130] | 106 [75, 130] | 0.03 |
| Respiratory rate, bpm, median [IQR] | | 16 [0, 22] | 16 [8, 24] | 0.09 |
| Body temperature, ℃, median [IQR] | | 36.0 [35.0, 36.5] | 36.0 [35.3, 36.5] | 0.04 |
| Glasgow Coma Scale, median [IQR] | | 3 [3, 11] | 3 [3, 12] | 0.06 |
| Abbreviations: OCCPR, open-chest cardiopulmonary resuscitation; OCCPR, closed-chest cardiopulmonary resuscitation; ASMD, Absolute standardized mean difference; IQR, interquartile range; AIS, abbreviated injury scale. | | | | |
